# Relationship Between Immunoinflammation and Coronary Physiology Evaluated by Quantitative Flow Ratio in Patients With Coronary Artery Disease

**DOI:** 10.3389/fcvm.2021.714276

**Published:** 2021-09-29

**Authors:** Chengzhe Liu, Zhiyao Yu, Huaqiang Chen, Jun Wang, Wei Liu, Liping Zhou, Yueyi Wang, Hu Chen, Huixin Zhou, Zhihao Liu, Jiapeng Han, Hong Jiang, Lilei Yu

**Affiliations:** ^1^Department of Cardiology, Renmin Hospital of Wuhan University, Wuhan, China; ^2^Cardiac Autonomic Nervous System Research Center of Wuhan University, Wuhan, China; ^3^Cardiovascular Research Institute, Wuhan University, Wuhan, China; ^4^Hubei Key Laboratory of Cardiology, Wuhan, China

**Keywords:** quantitative flow ratio, coronary physiology, coronary artery disease, immuno, inflammation

## Abstract

**Background:** The association between coronary physiology and immunoinflammation has not been investigated. We performed a retrospective study using quantitative flow ratio (QFR) to evaluate the interaction between immunoinflammatory biomarkers and coronary physiology.

**Methods:** A total of 172 patients with CAD who underwent coronary arteriography (CAG) and QFR were continuously enrolled from May 2020 to February 2021. As a quantitative indicator of coronary physiology, QFR can reflect the functional severity of coronary artery stenosis. The target vessel measured by QFR was defined as that with the most severe lesions. Significant coronary anatomical stenosis was defined as 70% stenosis in the target vessel.

**Results:** Compared with the QFR > 0.8 group, interleukin (IL)-6, IL-10, tumor necrosis factor (TNF)-α, and interferon (IFN)-γ were increased and CD3^+^ and CD4^+^ T lymphocyte counts were decreased in the QFR ≤ 0.8 group. In addition, patients with DS ≤ 70% had higher IL-6, IL-10, and TNF-α levels and decreased CD3^+^ and CD4^+^ T lymphocyte counts than those with DS > 70%. Logistic regression analysis indicated IL-6 to be an independent predictor of significant coronary functional and anatomic stenosis (odds ratio, 1.125; 95% CI, 1.059–1.196; *P* < 0.001). Receiver operating characteristic (ROC) analyses showed that IL-6 > 6.36 was predictive of QFR ≤ 0.8 of the target vessel. The combination of IL-6, IL-10 and CD4 improved the value for predicting QFR ≤ 0.8 of the target vessel (AUC, 0.737; 95% CI, 0.661–0.810).

**Conclusion:** Among immunoinflammatory biomarkers, IL-6 was independently associated with a higher risk of QFR ≤ 0.8 of the target vessel. The combination of immunoinflammatory biomarkers was highly predictive of significant coronary functional and anatomic stenosis.

## Introduction

Coronary artery disease (CAD) remains the most common cause of death worldwide, with atherosclerosis being the most common manifestation (1). Atherosclerosis is considered a chronic inflammatory disease, and both the immune system and inflammatory cells play a major role in its pathogenesis (2). The immune system influences the state of inflammatory cells by transforming them into proinflammatory or anti-inflammatory functional units and guiding interaction between different immune and inflammatory cells (3). Different inflammatory cells enter into the vascular wall and interact with each other to cause release of proinflammatory factors, which is a key step in the initiation and progression of plaque (4). Furthermore, plaque stability correlates with inflammatory cell levels. Immune cell-filled plaques are less stable and more prone to rupture, inducing major cardiovascular adverse events (5). A high level of inflammatory cytokines in CAD is closely associated with poor prognosis (6).

In addition, myocardial ischemia caused by substantial coronary stenosis has a strong influence on prognosis (3, 4), and physiological ischemia is important than anatomical stenosis in guiding the treatment of patients. In general, if coronary artery stenosis does not cause ischemia, the incidence of adverse cardiovascular events is low, and the prognosis is good (7, 8). Both increased inflammatory activity and the presence of coronary ischemia have a strong impact on the progression and prognosis of CAD. We hypothesized that markers of systemic immune function and inflammation are related to parameters reflecting the severity of coronary functional and anatomic stenosis.

Invasive fractional flow reserve (FFR) is currently considered the gold standard for evaluating the ischemic potential of CAD (8). However, due to the additional pressure guidewire requirements, invasive nature of the operation, and side effects of adenosine, the use of FFR in daily clinical practice is limited (9). QFR is a non-invasive angiographically derived FFR measurement. Previous studies have shown good agreement between measured values of QFR and FFR (10, 11), and QFR has a clear cutoff value for the diagnosis of coronary functional stenosis and demonstrates excellent reproducibility (11, 12). It has also been shown that QFR has high predictive value for prognosis (13, 14). Nevertheless, there is no evidence to date of the effects of immunity and inflammation on coronary physiology as detected by QFR in patients with CAD. In the current study, we evaluated the relationship between markers based on immune function and inflammatory activity and functional coronary lesions using QFR.

## Methods

### Study Population

**Figure 1:**
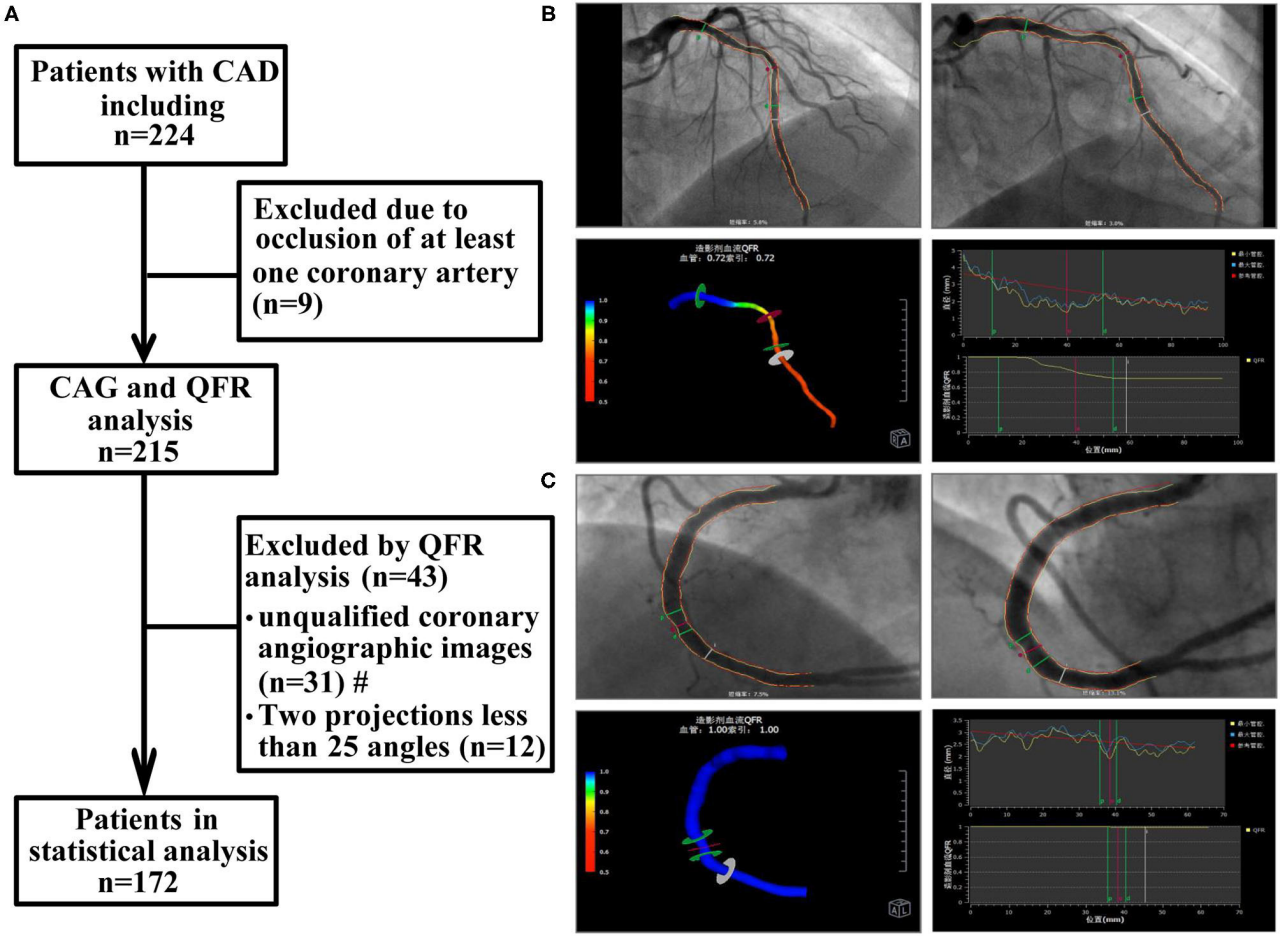
Study flow chart and QFR analysis process. **(A)** Study flow chart. **(B)** The QFR of LAD was calculated as 0.72. **(C)** The QFR of RCA was calculated as 1.00. The QFR of LAD, the smaller of the two, was selected as the QFR of the target vessel. CAD, coronary artery disease; CAG, coronary arteriography; QFR, quantitative flow ratio. #: unqualified coronary angiographic images included ostial lesion, severe vessel tortuosity, diffuse long lesions, and poor coronary image quality.

This retrospective observational study included 172 consecutive patients with CAD who underwent coronary arteriography (CAG) and QFR from May 2020 to March 2021 at the Department of Cardiology of Renmin Hospital of Wuhan University. CAD was diagnosed according to previously established guidelines (15). The main exclusion criteria were as follows: a diagnosis of ST-segment elevation myocardial infarction (STEMI), non-ST-segment elevation myocardial infarction (NSTEMI), coronary artery occlusion, or myocardial bridge; unqualified coronary angiographic images included ostial lesion, severe vessel tortuosity, diffuse long lesions, and poor coronary image quality; a lack of two images with a difference of more than 25 angles, overlap in the target lesion, excessive shortening or insufficient contrast agent filling, previous coronary artery bypass grafting (CABG); severe heart failure; cardiogenic shock; severe valvular disease; and previously known liver or kidney failure (estimated glomerular filtration rate <30 ml/min). In addition, patients with chronic inflammatory states, autoimmune disease, active infection, and malignancies were excluded. The flow chart for patient inclusion and exclusion is shown in Figure 1A. The study protocol was approved by the ethics committee of the Renmin Hospital of Wuhan University before patient enrollment.

### Coronary Angiography

All patients underwent CAG in accordance with standard procedures. Two independent cardiologists with clinical experience assessed the degree of stenosis of each coronary lesion. Consensus with a third investigator was indicated if disagreement occurred. Significant coronary anatomical stenosis was defined as 70% stenosis of the target vessel (16). Target vessels were defined as those with the most severe lesions.

### Computation of QFR

Offline QFR analysis was performed by a professional technician using the AngioPlus system (Pulse Medical Imaging Technology, Shanghai, China) following previously described procedures (10). Two angiographic images of the same coronary artery >25 degrees were selected for measurement. In the case of poor angiographic image quality, manual correction was performed according to standard operating procedures. Next, the contrast flow rate was obtained from coronary angiography images using the frame counting method, and then the contrast flow model was established for calculation. The QFR value obtained from the analysis was defined as the contrast-flow quantitative flow ratio (cQFR). Extensive studies have demonstrated that the cutoff value of QFR in coronary blood flow function is 0.8 (11, 17). For each patient, the coronary artery with the most clinically relevant or most severe stenosis was selected as the target vessel for subsequent analysis. The specific operation process is depicted in Figures 1B,C.

### Blood Collection and Laboratory Tests

Venous blood samples were collected from all patients before they underwent CAG. All samples were sent immediately to the hospital laboratory for direct testing to prevent any potential storage effects. The samples of all participants were subjected to routine whole blood analysis, including analysis of liver and kidney function, lipids, C-reactive protein (CRP), and N-terminal pro-brain natriuretic peptide (NT-pro-BNP). Concentrations of inflammatory cytokines, including IL-2, IL-4, IL-6, IL-10, TNF-α, and IFN-γ, were determined by flow cytometry (Becton Dickinson, FACSCalibur, USA) using a multiplex bead-based flow fluorescence immunoassay. Lymphocyte subsets were analyzed by flow cytometry (Becton Dickinson, FACSCanto II, USA). Additionally, multiple lymphocyte surface antigens were detected to distinguish different lymphocyte subsets (CD3^+^, CD4^+^, CD8^+^, CD19^+^, CD16^+^+56^+^); the number of cells in each subset was counted, and the value of CD4^+^/CD8^+^ was obtained. Levels of immunoglobulin (Ig)G, IgM, IgA, and complement C3 and C4 were measured by turbidimetric inhibition immunoassay using a specialized protein analysis system (IMMAGE 800 Automatic Special Protein Analyzer, Beckman, USA). A BN II protein meter (Siemens, Germany) was used to determine the concentration of IgE.

### Statistical Analysis

Categorical variables are represented by frequencies and percentages (%), and the frequencies of each index were compared using the chi-square test. Continuous variables are described in terms of the median and interquartile range (IQR) values. Unpaired *t*-tests and the Mann-Whitney test were used for comparisons, as appropriate. And correlation was determined by Pearson and Spearman rank analyses. Logistic regression analysis was also employed to evaluate independent predictors. Binary logistic regression analysis was used to obtain the prediction probability of single or multiple factors. ROC curve was drawn with the corresponding probability to analyze the prediction ability of each evaluation index. GraphPad Prism 7.0 software (GraphPad Software Inc.) and SPSS statistical software (version 26.0, IBM) were used for statistical analysis and chart drawing. *P* values <0.05 were considered significant.

## Results

### Baseline Characteristics

As presented in Table 1, the 172 patients included in the study were divided into two groups according to baseline measurements: target vessel QFR > 0.8 (*n* = 66) and ≤ 0.8 (*n* = 106). Compared with the QFR > 0.8 of the target vessel group, the proportion of male patients in the QFR ≤ 0.8 of the target vessel group was higher (54.5 vs. 78.8%, *p* = 0.006); CRP and NT-pro BNP, markers of inflammatory response and cardiac impairment, were significantly increased (both *p* = 0.001). The proportion of patients with QFR ≤ 0.8 who had ever received percutaneous coronary intervention (PCI) was higher than that in patients with QFR > 0.8 (51.5 vs. 25.5%, *p* = 0.001). However, there was no correlation between other indicators and QFR of the target vessel (Table 1).

**Table 1:** Baseline characteristics of QFR > 0.8 of the target vessel and QFR ≤ 0.8 of the target vessel.

**Patients**	**All subjects (*n* = 172)**	**QFR> 0.8 (*n* = 106)**	**QFR≤ 0.8 (*n* = 66)**	***P*-value**
Age, y	61.00 (53.00–68.00)	60.00 (52.00–68.00)	63.00 (55.00–69.00)	0.203
Male, %	114 (66.3)	62 (58.5)	52 (78.8)	0.006^*^
Current smoking, %	56 (32.6)	32 (30.2)	24 (36.4)	0.401
Family history of CAD, %	14 (8.1)	8 (7.5)	6 (9.1)	0.719
Diabetes mellitus, %	58 (33.7)	35 (33.0)	23 (34.8)	0.850
Hypertension, %	96 (55.8)	54 (50.9)	42 (63.6)	0.103
Hyperlipidemia, %	57 (33.1)	36 (34.0)	21 (31.8)	0.771
Previous MI, %	11 (6.4)	5 (4.7)	6 (9.1)	0.254
Previous PCI, %	61 (35.5)	27 (25.5)	34 (51.5)	0.001^*^
BMI, kg/m^2^	24.49 (22.70–26.35)	24.77 (22.75–26.57)	24.22 (22.49–26.12)	0.314
CRP, mg/L	2.60 (0.5–5.00)	1.50 (0.5–5.00)	5.00 (0.5–5.00)	0.001^*^
NT pro-BNP, pg/mL	65.21 (34.85–135.20)	53.51 (31.77–103.70)	88.77 (47.72–334.50)	0.001^*^
Total cholesterol, mmol/L	3.90 (3.13–4.86)	3.95 (3.14–4.98)	3.79 (3.07–4.72)	0.294
Triglyceride, mmol/L	1.50 (1.01–2.14)	1.49 (0.96–2.17)	1.50 (1.09–2.11)	0.82
HDL-c, mmol/L	0.99 (0.89–1.22)	1.01 (0.90–1.23)	0.98 (0.87–1.13)	0.204
LDL-c, mmol/L	2.18 (1.59–3.13)	2.17 (1.66–3.20)	2.21 (1.54–2.91)	0.583
Location of lesions vessel				0.872
LAD	91 (52.9)	55 (51.9)	36 (54.5)	
LCX	32 (18.6)	21 (19.8)	11 (16.7)	
RCA	49 (28.5)	30 (28.3)	19 (28.8)	

*Parameters were expressed as proportion and median IQR*.

* *A p-value < 0.05 was considered a statistically significant difference between the two groups*.

*QFR, quantitative flow ratio; BMI, body mass index; CAD, coronary artery disease; CRP, C-reactive protein; NT pro-BNP, N-terminal pro-B-type natriuretic peptide; HDL-c, High density lipoprotein-cholesterol; LDL, low density lipoprotein-cholesterol; MI, myocardial infarction; PCI, percutaneous coronary intervention; CABG, coronary artery bypass grafting; LAD, left anterior descending branch; LCX, left circumferential branch; RCA, right coronary artery; IQR, interquartile range*.

### Comparison of Immunoinflammatory Biomarkers Between Patients With QFR > 0.8 of the Target Vessel and QFR ≤ 0.8 of the Target Vessel

**Figure 2:**
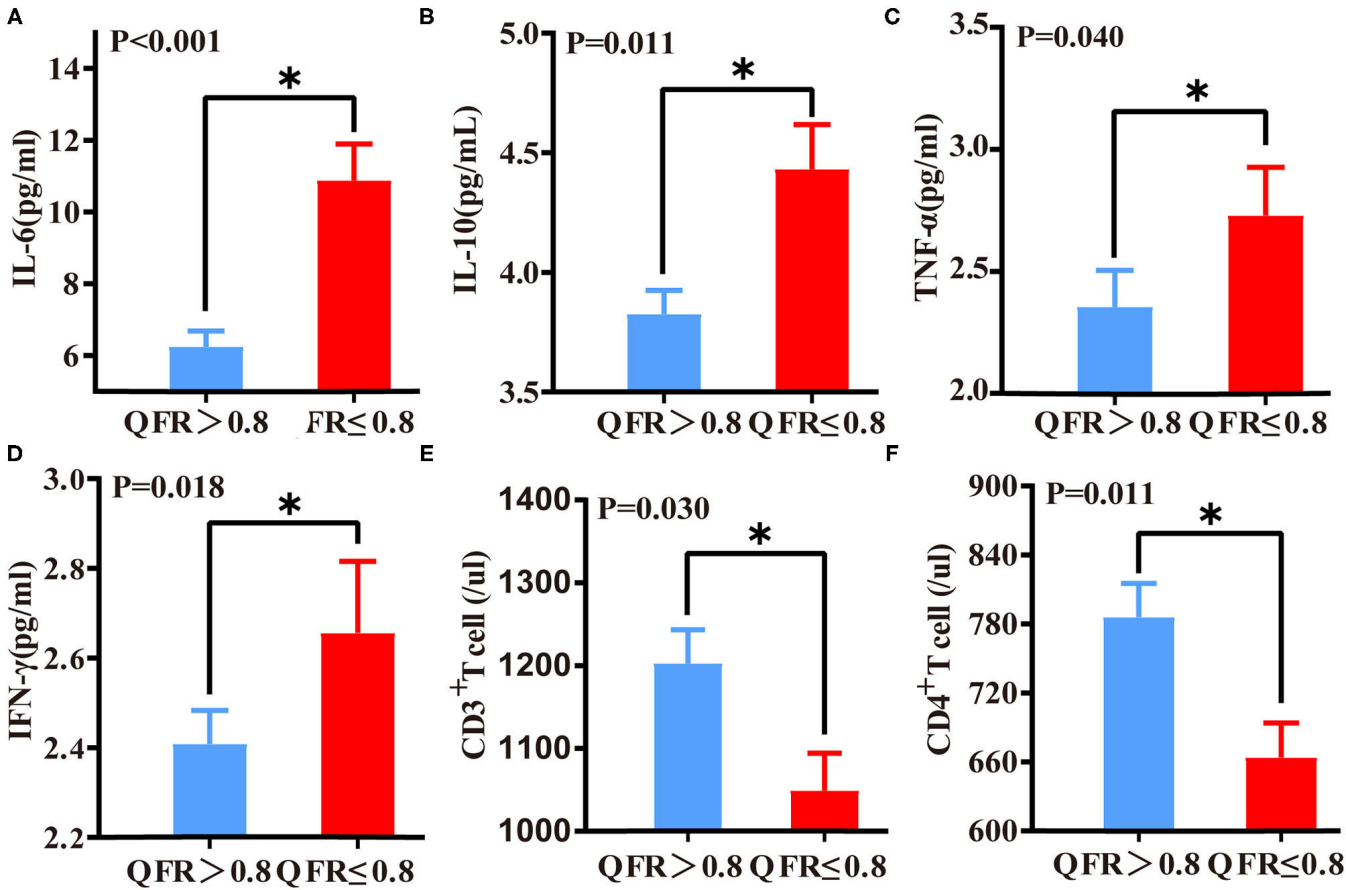
Differences in immunoinflammatory biomarkers between QFR > 0.8 of the target vessel and QFR ≤ 0.8 of the target vessel groups. Differences in IL-6 **(A)**, IL-10 **(B)**, TNF-α **(C)**, IFN-γ **(D)**, CD3+ T lymphocyte **(E)**, and CD4^+^ lymphocyte **(F)** levels between the QFR > 0.8 and QFR ≤ 0.8 groups. *A *p*-value < 0.05 was considered a statistically significant difference between the two groups.

The QFR ≤ 0.8 of the target vessel group showed statistically significant increases in levels of inflammatory factors, including IL-6 (*p* < 0.001), IL-10 (*p* = 0.011), TNF-α (*p* = 0.040), and IFN-γ (*p* = 0.018). However, CD3^+^ and CD4^+^ T lymphocytes were significantly reduced in patients with QFR ≤ 0.8 in the target vessel group (*p* = 0.015 and *p* = 0.011) (Figure 2). No other variables were significantly different (all *p* > 0.05).

### Correlations Between QFR and Immunoinflammatory Biomarkers

IL-6 (*r* = −0.386, *p* < 0.001) and IL-10 (*r* = −0.169, *p* = 0.027) correlated negatively with QFR. In contrast, biomarkers of immune function correlated positively with QFR. Among these biomarkers, CD4^+^ T lymphocytes (*r* = 0.225, *p* = 0.003) had a higher correlation with QFR than CD3^+^ T lymphocytes (*r* = 0.215, *p* = 0.005) (Supplementary Figure 1).

### Predictive Effects of Immunoinflammatory Biomarkers for QFR ≤ 0.8 of the Target Vessel

**Figure 3:**
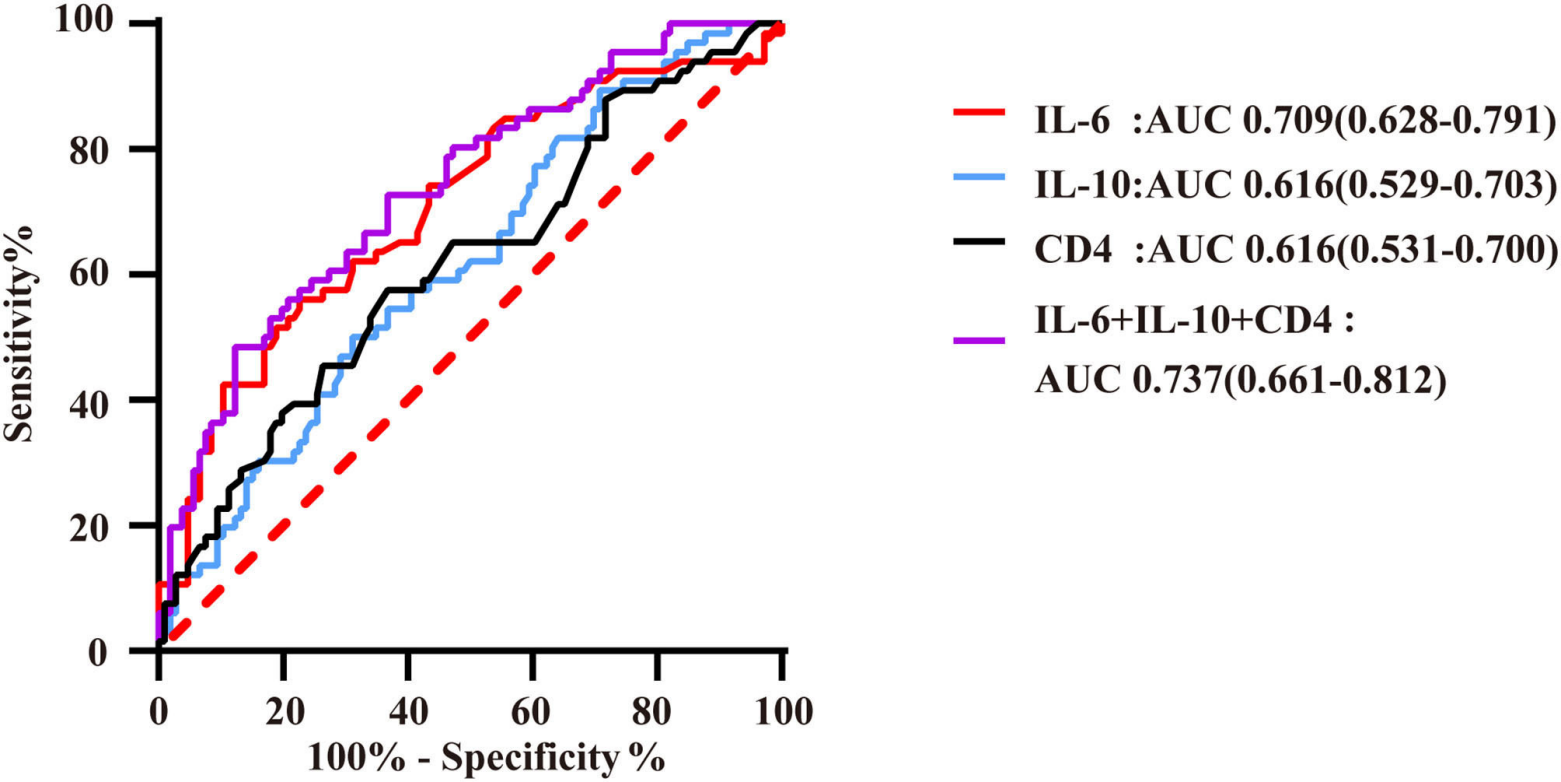
ROC curves of immunoinflammatory biomarkers predicting QFR ≤ 0.8 of the target vessel. AUC indicates the area under the ROC curve. The red curve represents the ROC curve of IL-6; the blue curve represents the ROC curve of IL-10; the black curve represents the ROC curve of CD4^+^ lymphocytes; and the purple curve represents the ROC curve of markers of immunoinflammatory association.

According to binary logistic regression analysis, IL-6, IL-10, and CD4^+^ T lymphocytes were predictors of QFR ≤ 0.8 of the target vessel. In addition, the significant predictive value of IL-6 was maintained after adjusting for traditional variables associated with coronary ischemia (Table 2). The CD3^+^ T lymphocyte count represents the number of all T cells, including CD4^+^ T lymphocytes and CD8^+^ T lymphocytes. As CD4^+^ T lymphocytes correlated more strongly with QFR than CD3^+^ T lymphocytes (Figure 3), we substituted CD4^+^ T lymphocytes for CD3^+^ T lymphocytes.

**Table 2:** Logistic regression analyses of immunoinflammatory biomarker levels for QFR ≤ 0.8 of the target vessel.

**Factor**	**Unadjusted**		**Model 1**		**Model 2**		**Model 3**	
	**Odds ratio (95%CI)**	***p*-value**	**Odds ratio (95% CI)**	***p*-value**	**Odds Ratio (95% CI)**	***p*-value**	**Odds Ratio (95% CI)**	***p*-value**
IL-6	1.126 (1.062–1.194)	<0.001^*^	1.116 (1.055–1.182)	<0.001^*^	1.116 (1.052–1.183)	<0.001^*^	1.125 (1.059–1.196)	<0.001^*^
IL-10	1.429 (1.103–1.852)	0.007^*^	1.230 (0.929–1.630)	0.149	1.189 (0.890–1.588)	0.242	1.133 (0.838–1.531)	0.417
CD4	0.998 (0.997–0.999)	0.045^*^	0.998 (0.996–1.000)	0.016^*^	0.998 (0.997–1.000)	0.031^*^	0.999 (0.997–1.000)	0.110

*Odds ratio shown were for immuno-inflammatory biomarker level as a continuous variable*.

* *A p-value <0.05 was considered significant for statistical significance*.

*Unadjusted model performed univariate regression analysis on biomarkers. Model 1 put IL-6, IL-10, CD4 and together for multivariate regression analysis. Model 2 adjusted for NT pro-BNP and CRP. Model 3 adjusted for all factors in model 2 plus age, sex, BMI, hypertension, diabetes mellitus, smoking, and blood lipids*.

**Figure 4:**
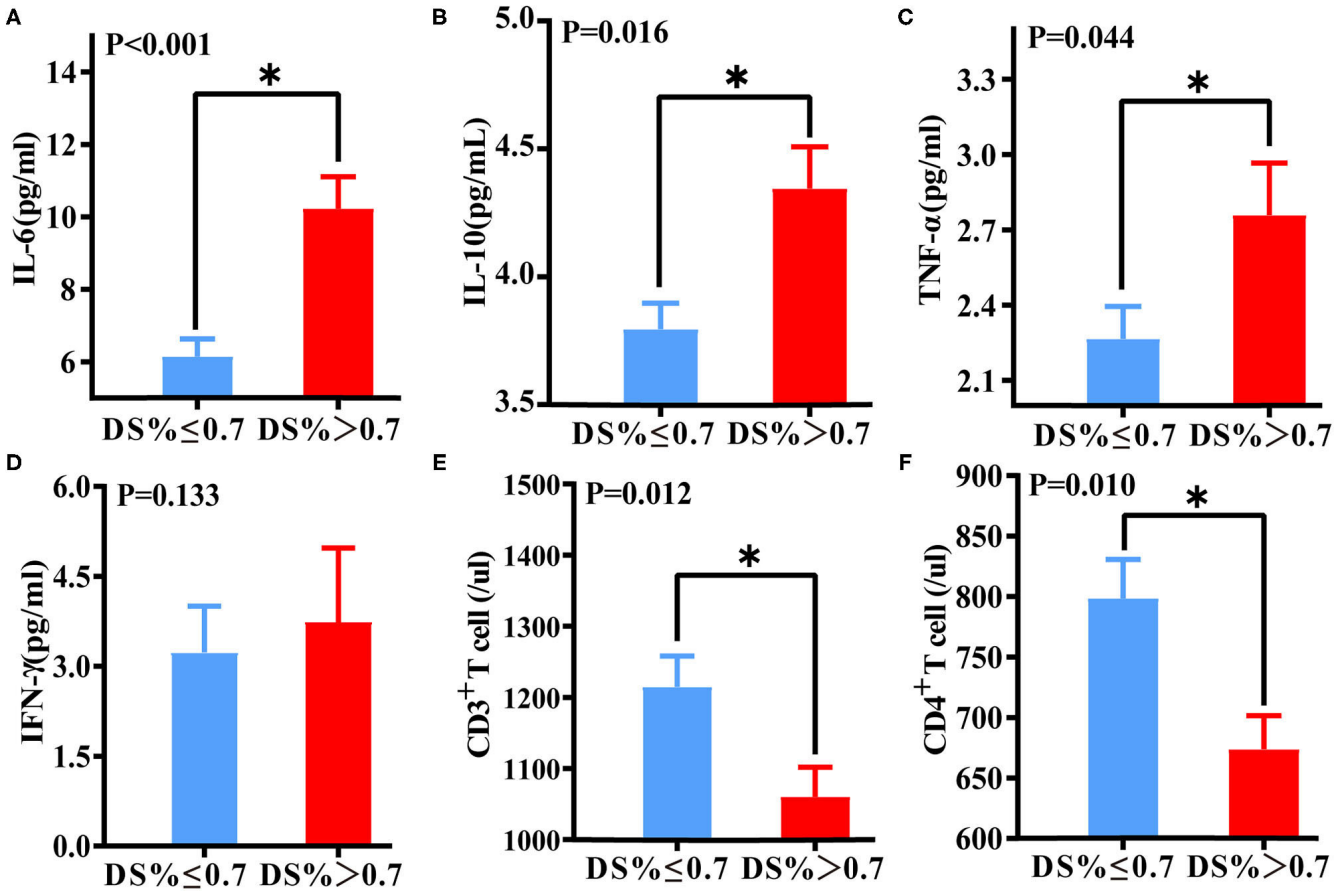
Differences in immune function and inflammatory biomarkers between DS > 70% of the target vessel and DS ≤ 70% of the target vessel groups. Differences in IL-6 **(A)**, IL-10 **(B)**, TNF-α **(C)**, IFN-γ **(D)**, CD3^+^ T lymphocyte **(E)**, and CD4^+^ lymphocyte **(F)** levels between the DS > 70% and DS ≤ 70% groups. *A *p*-value < 0.05 was considered a statistically significant difference between the two groups.

IL-6 had high predictive value for QFR ≤ 0.8 of the target vessel, with an area under the ROC curve of 0.709 (95% CI: 0.628–0.791). Moreover, the number of CD4^+^ T lymphocytes and IL-10 level had a certain predictive value for QFR, with AUCs of 0.616 (95% CI: 0.531–0.700) and 0.616 (95% CI: 0.529–0.703), respectively. In this study, the combination of these biomarkers resulted in a higher predictive value for QFR ≤ 0.8 of the target vessel, with an area under the ROC curve that increased to 0.737 (95% CI: 0.661–0.812) (Figure 4). The cutoff threshold of IL-6 to generate the maximum summation of sensitivity and specificity in discriminating QFR ≤ 0.8 of the target vessel was 6.36; the corresponding sensitivity and specificity were 56.1 and 77.4%, respectively. In addition, the cutoff value of the combination of immunoinflammatory biomarkers (IL-6, IL-10 and CD4) was associated with 48.5% sensitivity and 88.7% specificity (Supplementary Table 1).

### Association of Immunoinflammatory Biomarkers With Coronary Target Vessel Lesions

Based on the results of the corresponding CAG, the patients were grouped according to the 70% degree of coronary artery diameter stenosis (DS) of the target vessel. The proportions of male patients and patients who underwent PCI, as well as CRP and BNP levels, were significantly higher among those with DS > 70% than those with DS ≤ 70% (*p* = 0.001, *p* < 0.001, *p* = 0.004, *p* = 0.008, respectively). Compared with the DS ≤ 70% of the target vessel group, the DS > 70% of the target vessel group had lower levels of total cholesterol (*p* = 0.045) and high-density lipoprotein (HDL) (*p* = 0.034). There was no significant difference between other indicators and DS of the target vessel (Supplementary Table 2).

**Figure 5:**
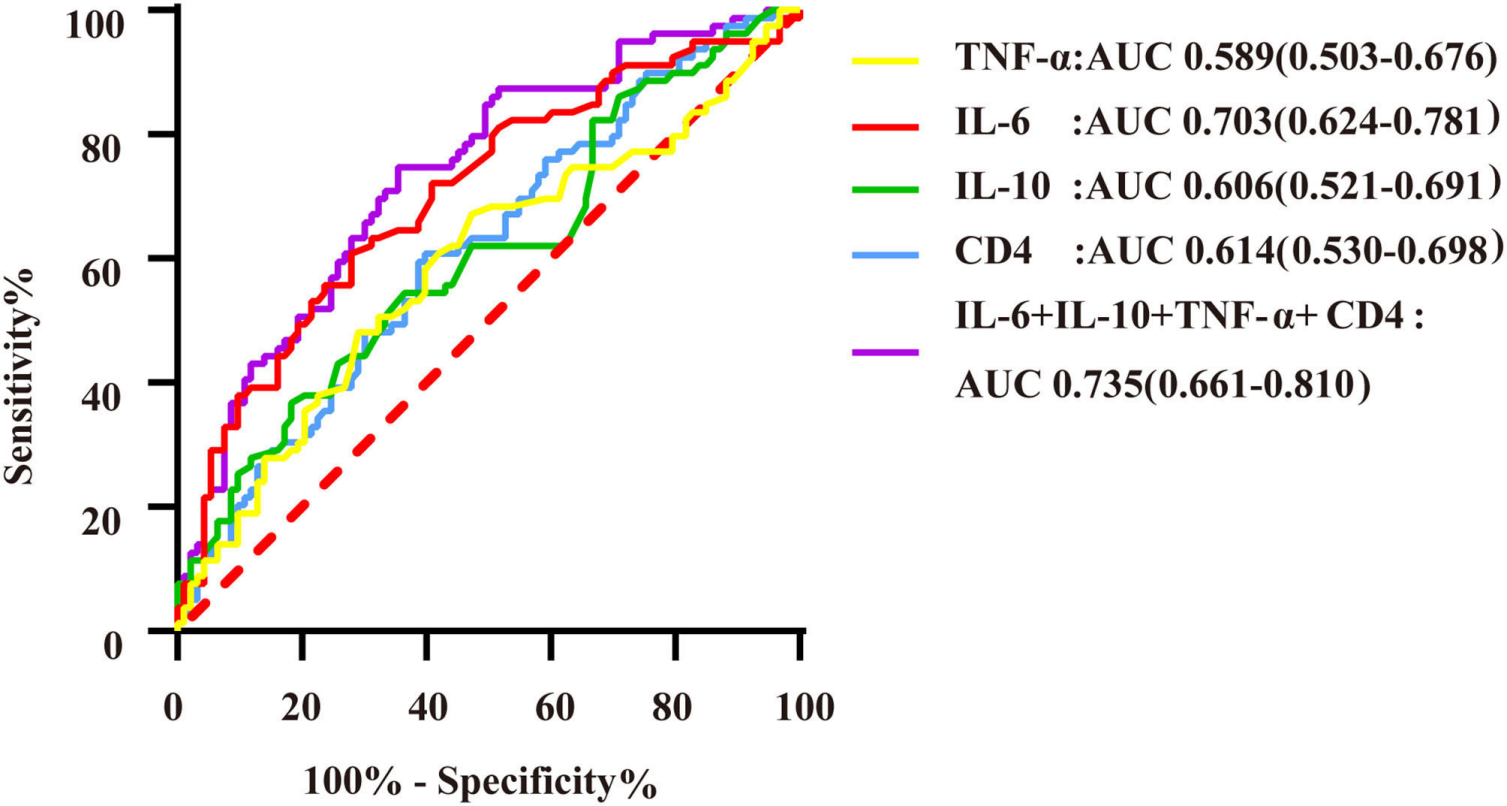
ROC curves of immunoinflammatory biomarkers predicting DS > 70% of the target vessel. AUC indicates the area under the ROC curve. The yellow curve represents the ROC curve of TNF-α; the red curve represents the ROC curve of IL-6; the green curve represents the ROC curve of IL-10; the blue curve represents the ROC curve of CD4^+^ T lymphocytes; and the purple curve represents the ROC curve of markers of immuno-inflammatory association.

Levels of IL-6 (*P* < 0.001), IL-10 (*p* = 0.016), and TNF-α (*P* = 0.044) in the DS > 70% target vessel group were much higher than those in the DS ≤ 70% target vessel group. Conversely, levels of CD3^+^ T lymphocytes (*P* = 0.012) and CD4^+^ T lymphocytes (*P* = 0.010) in the DS > 70% of the target vessel group were significantly lower than those in the DS ≤ 70% of the target vessel group (Figure 5).

Logistic regression analysis showed that IL-6, IL-10, and TNF-α levels and the CD4^+^ T lymphocyte count were able to predict DS > 70% of the target vessel. According to multivariate regression analysis, IL-6 was an independent predictor of DS > 70% of the target vessel (Supplementary Table 3). ROC curve analysis indicated that the combination of IL-6, IL-10, TNF-α, and CD4^+^ T lymphocyte biomarkers had a high predictive value for DS > 70% of the target vessel, with an AUC of 0.735 (95% CI, 0.661–0.810) (Figure 5). In addition, the cutoff value of IL-6 (5.62) was associated with 60.8% sensitivity and 72% specificity, with that of the combination of immunoinflammatory biomarkers (0.403) related to 73.4% sensitivity and 62.4% specificity (Supplementary Table 4).

## Discussion

This study suggests that specific biomarkers of immune function and inflammatory factors, which are traditional biomarkers, are independent predictors of significant coronary anatomical and functional stenosis in patients with CAD. High levels of IL-6 and IL-10 and low CD4^+^ T lymphocyte counts were significantly associated with decreased coronary hemodynamics (QFR ≤ 0.8). Moreover, a combination of these biomarkers was superior to the individual markers in predicting functional coronary stenosis, with the largest area under the ROC curve (Figure 4). Furthermore, this study shows that these biomarkers have a certain predictive value for coronary artery anatomic stenosis.

### Inflammation and Coronary Physiology

The relationship between inflammation and atherosclerosis is well-established, and various immune system cells and inflammatory factors are known to play a crucial role in the initiation and progression of CAD (1, 2). However, the link between inflammation and coronary physiology remains unclear. Our results are consistent with previous studies showing that inflammatory markers used for CAD focus on downstream inflammatory markers such as CRP (18). Upstream inflammatory markers, such as proinflammatory factors, have recently been suggested to control the inflammatory cascade, which may be more directly related to CAD (19). A large number of studies have shown that IL-6 plays a key role in the progression of coronary atherosclerosis (20, 21). IL-6 promotes the proliferation of neutrophils and monocytes as well as expression of adhesion molecules by vascular endothelial cells to enhance the local inflammatory response (22). In addition, IL-6 induces liver cells to synthesize fibrinogen and CRP (23). IL-6 correlated independently with functional and anatomic coronary stenosis in our study. Tocilizumab, an inhibitor of the IL-6 receptor, is being analyze in clinical trials with respect to its therapeutic effect and mechanism in CAD (24, 25). Tocilizumab may affect the prognosis of CAD not only by inhibiting inflammation but also by directly regulating the severity of coronary physiology. The mechanism deserves further investigation.

Numerous studies have shown that IL-10 is a cytokine secreted mainly by monocyte macrophages and lymphocytes with inflammatory protective effects (26). Interestingly, our results revealed increased levels of IL-10 in a group of patients with more severe functional (*p* = 0.011) and anatomical (*p* = 0.016) coronary stenosis, and even elevated IL-10 levels were somewhat predictive of significant functional and anatomical coronary lesions. Similarly, some studies have reported a long-term increase in serum IL-10 levels in patients with unstable CAD or myocardial infarction compared to levels in healthy subjects or those with stable CAD (27, 28), and IL-10 levels are higher in high-risk patients with atherosclerosis (29). We speculate that elevated IL-10 may be associated with inflammatory activation of local coronary vessels or the immune system. In addition, increased IL-10 levels can reduce the inflammatory response in coronary plaques, stabilize plaques and improve the prognosis of patients with CAD (30). In the CAPTURE trial, elevated serum IL-10 levels correlated with significant improvement in prognosis in those with acute coronary syndrome (ACS) (31).

### Immunity and Coronary Physiology

A low lymphocyte count is a common manifestation of the inflammatory response, and both basic and clinical studies have suggested that a low lymphocyte count plays an important role in the progression of CAD (32, 33). The direct effectors that play a major role in inflammation are neutrophils and monocytes, whereas lymphocytes play a more extensive role in the regulation of an inflammatory response at various stages of atherosclerosis (34). Reduced lymphocyte counts are also associated with poor prognosis in patients with a variety of cardiovascular diseases, such as stable CAD (35) and ACS, including STEMI and NSTEMI (36–38), and unstable angina pectoris (39). In this study, the numbers of CD3^+^ lymphocytes (indicating the total number of lymphocytes) and CD4^+^ lymphocytes (indicating the number of CD4+ T lymphocytes) were significantly reduced in patients with QFR ≤ 0.8 of the target vessel. At present, it is believed that the lymphocyte number may be reduced for the following reasons. On the one hand, lymphocyte apoptosis is increased in the inflammatory state. Under pathological conditions, inflammation indiscriminately damages lymphocytes, including Th1 cells (proinflammatory CD4^+^ lymphocytes) and Th2 cells (anti-inflammatory CD4^+^ lymphocytes). In the presence of uncleared phagocytic apoptotic cells, some heat shock proteins that interact with Toll-like receptors (TLRs) are released through cell lysis, thereby promoting secretion of proinflammatory factors by macrophages. There is a vicious cycle between the inflammatory cascade and immune cell apoptosis (40). On the other hand, patients with CAD are in a state of systemic stress response, during which the secretion of serum cortisol and catecholamine increases, which may directly lead to a decrease in lymphocyte count (41).

### The Relationship Between Immunity, Inflammation and FFR

Previous studies on the link between inflammation and FFR have produced similar results. Versteeg et al. (42) demonstrated that TLRs, which play a key role in innate immunity, are significantly associated with stenosis of the tube diameter, the number of disaffected vessels, and FFR outcomes. In addition, Erdogan et al. (43) found that an increase in the number of inflammatory cells and a decrease in the number of lymphocytes had a certain predictive power for FFR in patients with chronic coronary syndrome. In the present study, we demonstrated that immunoinflammatory biomarkers are closely related to coronary artery functional and anatomical stenosis. Therefore, our data confirm that immunoinflammatory biomarkers can serve as a therapeutic target in atherosclerosis (44, 45) and appear to be independent of and at least as powerful as traditional risk factors for myocardial infarction and atherosclerosis (46).

### Limitations

This study has some limitations. First, this study involved a retrospective and observational design with a limited sample size. Second, follow-up data and endpoint event analysis were lacking. Third, other inflammatory markers, such as high-sensitivity CRP, were not included in the analysis because parameters with missing data were not included. Fourth, a gold standard control (FFR) was not included. Fifth, the QFR data were derived from the most severe stenosis observed in each of the main coronary vessels assessed. Thus, our study may not have fully taken the overall flow of the patient's coronary arteries into account, and further analysis should be performed in future well-designed clinical trials.

## Conclusions

Inflammatory factors and immune function are associated with coronary artery anatomic stenosis and functional ischemia. IL-6 was found to be the most significant independent predictor of functional coronary artery stenosis detected through QFR. Furthermore, the combination of IL-6, IL-10, and CD4^+^ T lymphocytes is a better predictor of functional coronary stenosis than any single biomarker. The combination of multiple indicators significantly increases the probability of identifying functional severe coronary artery disease, which is worthy of investigation in a broader and more specific state of heart disease.

## Data Availability Statement

The original contributions presented in the study are included in the article/Supplementary Material, further inquiries can be directed to the corresponding author/s.

## Ethics Statement

Ethical review and approval was not required for this study with human participants, in accordance with the local legislation and institutional requirements.

## Author Contributions

LY and HJ: substantial contributions to conception and design, data acquisition, or data analysis, and interpretation. CL, ZY, HuaC, JW, WL, ZL, YW, HuC, HZ, ZL, and JH: drafting the article or critically revising it for important intellectual content. CL, ZY, HuC, JW and WL: final approval of the version to be published and agreement to be accountable for all aspects of the work in ensuring that questions related to the accuracy or integrity of the work are appropriately investigated and resolved. All authors contributed to the article and approved the submitted version.

## Funding

This work was supported by the National Key R&D Program of China [2017YFC1307802], and the National Natural Science Foundation of China [81970287, 81530011, 81770364, 81871486, and 82100530].

## Conflict of Interest

The authors declare that the research was conducted in the absence of any commercial or financial relationships that could be construed as a potential conflict of interest.

## Publisher's Note

All claims expressed in this article are solely those of the authors and do not necessarily represent those of their affiliated organizations, or those of the publisher, the editors and the reviewers. Any product that may be evaluated in this article, or claim that may be made by its manufacturer, is not guaranteed or endorsed by the publisher.
