# Supplementary material for: Relationship Between Immunoinflammation and Coronary Physiology Evaluated by Quantitative Flow Ratio in Patients With Coronary Artery Disease
Source: Front Cardiovasc Med. 2021 Sep 29;8:714276. doi: 10.3389/fcvm.2021.714276 (PMC8511462; doi:10.3389/fcvm.2021.714276)
Supplement: Supplementary file 1 [file Data_Sheet_1.docx]

| **Supplementary table 1** Diagnostic Performance of Immuno-inflammatory Biomarkers in Distinguishing QFR ≤ 0.8 of the target vessel. | | | | | | | |
| --- | --- | --- | --- | --- | --- | --- | --- |
| factor | AUC | 95%CI | SE | p value | Sensitivity | Specificity | Cutoff Value |
| IL-6 | 0.710 | 0.628-0.791 | 0.041 | <0.001* | 56.1% | 77.4% | 6.36 |
| IL-10 | 0.616 | 0.529-0.703 | 0.044 | 0.011* | 57.6% | 63.2% | 3.785 |
| CD4 | 0.616 | 0.531-0.700 | 0.043 | 0.011* | 68.9% | 50% | 638 |
| IL-6+CD4+IL-10 | 0.737 | 0.661-0.812 | 0.039 | <0.001* | 48.5% | 88.7% | 0.457# |
| #Optimal threshold value obtained from the data, which was the threshold leading to the maximum summation of sensitivity and specificity.  *p-values < 0.05 are considered significant for statistical significance.  AUC, area under the ROC curve; CI, confidence interval; SE, standard error. | | | | | | | |

| Supplementary table 2 Baseline characteristics of patients with the target vessel stenosis ≤ 70% and patients with the target vessel stenosis > 70%. | | | | |
| --- | --- | --- | --- | --- |
| Patients | All subjects(n=172) | DS%≤ 70%(n=97) | DS%> 70%(n=75) | P value |
| Age, y | 61.00(53.00-68.00) | 59.00(51.00-68.00) | 62.50(55.00-68.75) | 0.063 |
| Men, % | 114(66.3) | 54(55.7) | 60(80.0) | 0.001* |
| Current smoking, % | 56(32.6) | 30(30.9) | 26(34.7) | 0.604 |
| Family history of CAD, % | 14(8.1) | 8(8.2) | 6(8.0) | 0.953 |
| Diabetes mellitus, % | 58(33.7) | 32(33.0) | 26(34.7) | 0.818 |
| Hypertension, % | 96(55.8) | 49(50.5) | 47(62.7) | 0.112 |
| Hyperlipidemia, % | 57(33.1) | 32(33.0) | 25(33.3) | 0.962 |
| Previous MI, % | 11(6.4) | 3(3.1) | 8(10.7) | 0.044* |
| Previous PCI, % | 61(35.5) | 23(23.7) | 38(50.7) | ＜0.001* |
| BMI, kg/m^2^ | 24.49(22.70-26.35) | 24.54(22.70-26.78) | 24.45(22.55-26.08) | 0.495 |
| CRP, mg/L | 2.60(0.5-5.00) | 1.40(0.5-5.00) | 5.00(0.5-5.00) | 0.004* |
| NT pro-BNP, pg/mL | 65.21(34.85-135.20) | 54.01(31.83-106.50) | 76.28(43.45-241.55) | 0.008* |
| Total cholesterol, mmol/L | 3.90(3.13-4.86) | 4.14(3.19-5.03) | 3.74(3.03-4.54) | 0.045* |
| Triglyceride, mmol/L | 1.50(1.01-2.14) | 1.48(0.98-2.19) | 1.51(1.02-2.06) | 0.967 |
| HDL-c, mmol/L | 0.99(0.89-1.22) | 1.03(0.92-1.25) | 0.97(0.86-1.13) | 0.034* |
| LDL-c, mmol/L | 2.18(1.59-3.13) | 2.37(1.70-3.21) | 2.14(1.49-2.82) | 0.083 |
| Location of lesions vessel |  |  |  | 0.702 |
| LAD | 91(52.9) | 52(53.6) | 39(52.0) |  |
| LCX | 32(18.6) | 16(16.5) | 16(21.3) |  |
| RCA | 49(28.5) | 29(29.9) | 20(26.7) |  |
| Parameters were expressed as proportion and median IQR.  *p-value < 0.05 was considered a statistically significant difference between the two groups.  DS, diameter stenosis; BMI, body mass index; CAD, coronary artery disease; CRP, C-reactive protein; NT pro-BNP, N-terminal pro-B-type natriuretic peptide; HDL-c, High density lipoprotein-cholesterol; LDL, low density lipoprotein-cholesterol; MI, myocardial infarction; PCI, percutaneous coronary intervention; CABG, coronary artery bypass grafting; LAD, left anterior descending branch; LCX, left circumferential branch; RCA, right coronary artery; IQR, interquartile range. | | | | |

| **Supplementary table 3** Logistic Regression Analyses of Immuno-inflammatory Biomarkers Levels for DS > 70% of the target vessel. | | | | | | | | | |
| --- | --- | --- | --- | --- | --- | --- | --- | --- | --- |
| factor | Unadjusted | | Model 1 | | Model 2 | | | Model 3 | |
|  | Odds Ratio  (95%CI) | p value | Odds Ratio  (95%CI) | p value | Odds Ratio  (95%CI) | p value | Odds Ratio  (95%CI) | | p value |
| IL-6 | 1.120  (1.053-1.191) | <0.001* | 1.120  (1.055-1.188) | 0.001* | 1.117  (1.047-1.192) | 0.001* | 1.125  (1.053-1.202) | | 0.001* |
| IL-10 | 1.465  (1.117-1.923) | 0.006* | 1.231  (0.921-1.645) | 0.150 | 1.163  (0.856-1.581) | 0.334 | 1.119  (0.818-1.530) | | 0.482 |
| TNF-α | 1.244  (1.001-1.544) | 0.049* | 0.360  (0.144-0.898) | 0.283 | 1.187  (0.923-1.527) | 0.181 | 1.161  (0.911-1.480) | | 0.226 |
| CD4 | 0.998  (0.997-1.000) | 0.024* | 0.998  (0.996-1.000) | 0.021* | 0.998  (0.997-1.000) | 0.019* | 0.999  (0.997-1.000) | | 0.100 |
| Odds ratio shown were for immuno-inflammatory biomarker level as a continuous variable.  *p-values < 0.05 are considered significant for statistical significance.  Unadjusted model performed univariate regression analysis on biomarkers, and Model 1 put IL-6, IL-10, TNF-α and CD4 together for multivariate regression analysis. Model 2 adjusted for NT pro-BNP and CRP. Model 3 adjusted for all factors in model 2 plus age, sex, BMI, hypertension, diabetes mellitus, smoking, and blood lipids. | | | | | | | | | |

| **Supplementary table 4** Diagnostic Performance of Immuno-inflammatory Biomarkers in Distinguishing DS > 70% of the target vessel. | | | | | | | |
| --- | --- | --- | --- | --- | --- | --- | --- |
| factor | AUC | 95%CI | SE | p value | Sensitivity | Specificity | Cutoff Value |
| IL-6 | 0.703 | 0.624-0.781 | 0.040 | <0.001* | 60.8% | 72% | 5.62 |
| IL-10 | 0.606 | 0.521-0.691 | 0.043 | 0.017* | 36.7% | 81.7% | 4.185 |
| CD4 | 0.614 | 0.530-0.698 | 0.043 | 0.010* | 60.2% | 60.8% | 700 |
| TNF-α | 0.589 | 0.503-0.676 | 0.044 | 0.044* | 67.1% | 52.7% | 1.88 |
| IL-6+CD4+  IL-10+ TNF-α | 0.737 | 0.661-0.812 | 0.039 | <0.001* | 73.4% | 62.4% | 0.403# |
| #Optimal threshold value obtained from the data, which was the threshold leading to the maximum summation of sensitivity and specificity.  *p-values < 0.05 are considered significant for statistical significance.  AUC, area under the ROC curve; CI, confidence interval; SE, standard error. | | | | | | | |
