# Supplementary figures and images for: Relationship Between Immunoinflammation and Coronary Physiology Evaluated by Quantitative Flow Ratio in Patients With Coronary Artery Disease
Source: Front Cardiovasc Med. 2021 Sep 29;8:714276. doi: 10.3389/fcvm.2021.714276 (PMC8511462; doi:10.3389/fcvm.2021.714276)

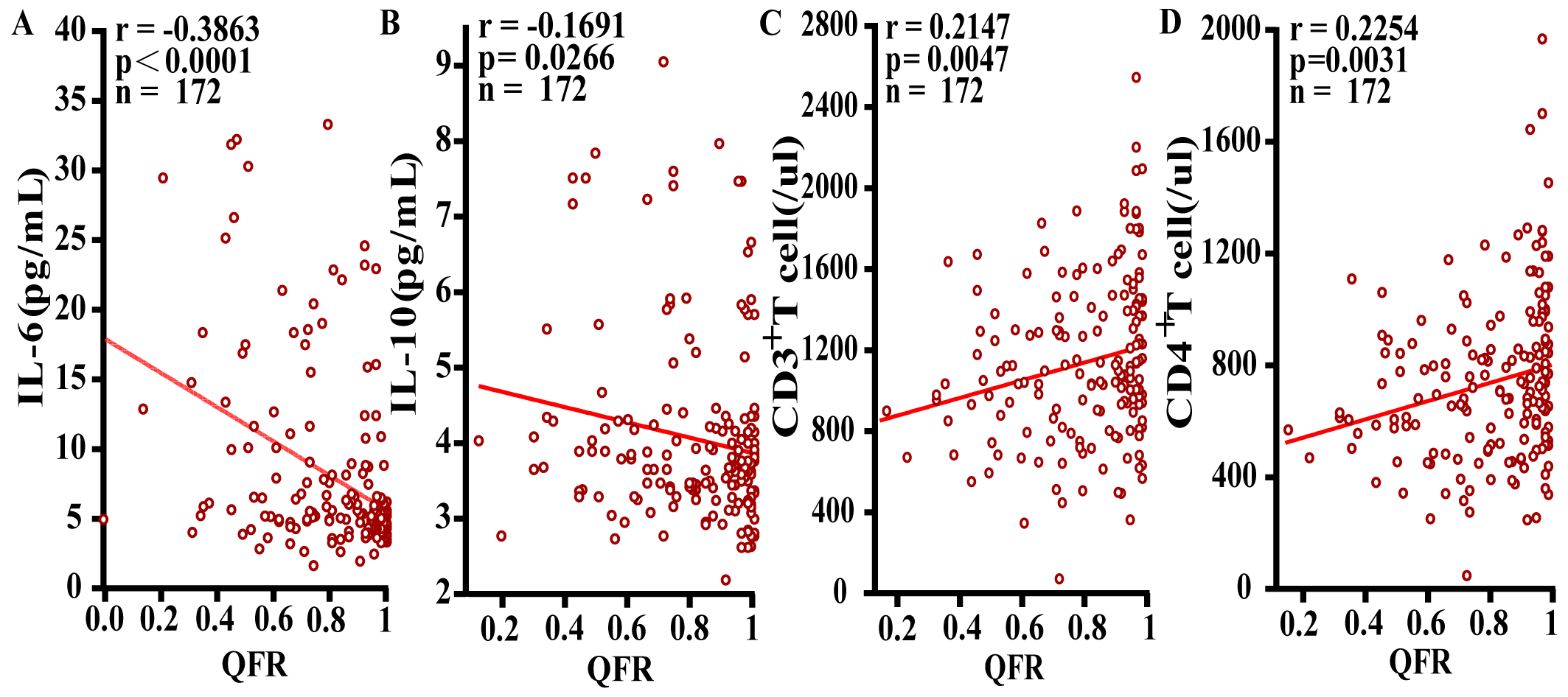

Supplement: Supplementary file 2 [file Image_1.TIF]
